# Management of an obstructed hepatoportocholecystostomy via an endoscopic cholecystoduodenostomy

**DOI:** 10.1055/a-2474-7635

**Published:** 2024-12-10

**Authors:** Gabriel Marcellier, Paul Rivallin, Abdellah Hedjoudje, Kenza Bourhrara, Benedicte Jais, Ryad Smadhi, Frederic Prat

**Affiliations:** 1 55100 Endoscopy Unit, Hopital Beaujon, Clichy, France; 2 37055 Gastroenterology Unit, Centre Hospitalier Compiegne-Noyon, Compiegne, France

Biliary atresia is a rare perinatal disease leading to the child’s death if left untreated 1 . There are several types of biliary atresia depending on the remaining functional biliary tract 2 . The most common (75%) is type 4 with complete extrahepatic atresia; here the Kasai intervention is indicated, consisting of an anastomosis between the hepatic hilum and a Roux-en-Y loop ( Fig. 1 **a** ) 3 4 . In some cases (type 3, 20%), the gallbladder and cystic duct are still patent and an hepatoportocholecystostomy (or “gallbladder–Kasai” surgery) can be performed. The hilar plate is then stitched to the gallbladder, which reduces the risk of cholangitis ( Fig. 1 **b** ) 5 .

**Fig. 1:**
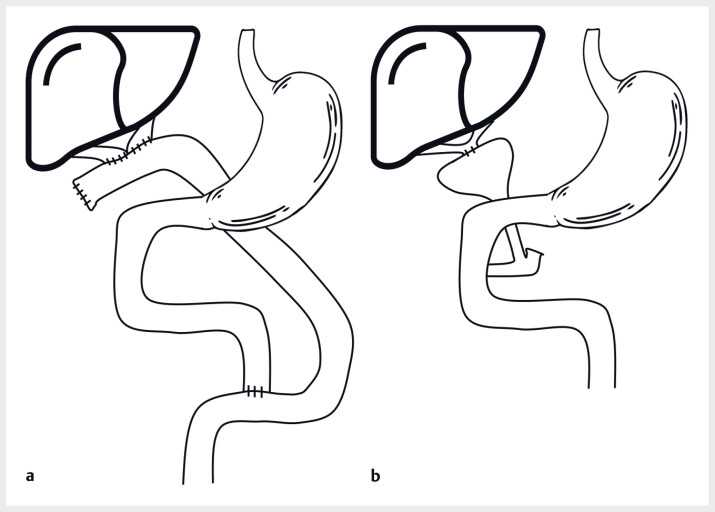
Kasai and gallbladder–Kasai surgery: **a** standard Kasai procedure with an hepatoportoenterostomy where the hepatic hilum is anastomosed with a Roux-en-Y jejunal loop; **b** gallbladder–Kasai surgery with an anastomosis between the hilar plate and the gallbladder.

We present the case of a patient who had undergone gallbladder–Kasai surgery in the 1980s and developed jaundice related to a stricture of the gallbladder–hilum anastomosis ( Video 1 ).

Endoscopic management of a strictured gallbladder–hilum anastomosis in a patient with gallbladder–Kasai anatomy.Video 1

We first attempted to perform a retrograde transpapillary cannulation of the cystic duct. After dilating several strictures, we accessed the gallbladder, but were unable to enter the anastomosis ( Fig. 2 **a** ).

**Fig. 2:**
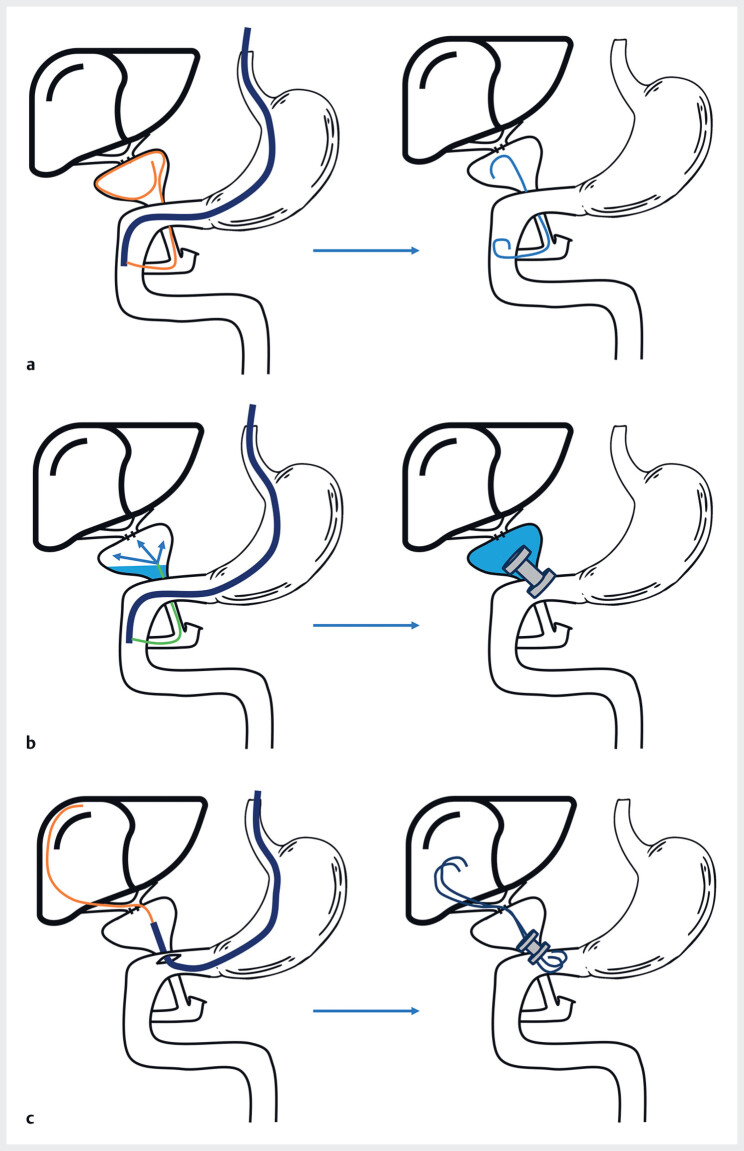
Endoscopic intervention steps. **a** Retrograde transpapillary cannulation of the cystic duct and dilation of several strictures. A double-pigtail stent was positioned between the gallbladder and the duodenum. **b** A few weeks later, after filling the gallbladder, a cholecystoduodenostomy was created with a 10 × 15-mm lumen-apposing metal stent (LAMS). **c** At a further session some weeks later, after removal of the LAMS, a gastroscope was used to enter the gallbladder and catheterize the hilar anastomosis. The anastomosis was dilated to 6 mm and two 7-Fr/5-cm double-pigtail stents were inserted. The same LAMS was reintegrated into the operating channel of the gastroscope and repositioned to maintain the cholecystoduodenal anastomosis.

We subsequently decided to perform a cholecystoduodenostomy a few weeks later using a lumen-apposing metallic-stent (LAMS), allowing access to the anastomosis by entering the gallbladder directly. The procedure was done under endoscopic ultrasound (EUS) guidance, using a 10 × 15-mm LAMS ( Fig. 2 **b** ). Despite several attempts, we were unable to directly cannulate the stricture. Further treatment for the patient was therefore rescheduled for a few weeks later. After removal of the LAMS, which was obstructing visibility, we were able to enter the gallbladder with a gastroscope and to cannulate and dilate the anastomosis. Two 7-Fr/5-cm double-pigtail stents were then inserted ( Fig. 2 **c** ). The same LAMS was then reintegrated into the operating channel and repositioned, to maintain the cholecystoduodenostomy in case endoscopic revisions should be required.

At 1 month after the procedure, computed tomography confirmed correct stent positioning and also aerobilia. The patient’s liver function returned to normal.

To our knowledge, this is the first case describing cholecystoduodenostomy to treat a strictured gallbladder–Kasai anastomosis. This procedure was complication-free and efficient. It illustrates the effectiveness of combining different interventional techniques to address complex cases.

Endoscopy_UCTN_Code_TTT_1AS_2AH

## Notes

Endoscopy E-Videos https://eref.thieme.de/e-videos
*E-Videos* is an open access online section of the journal *Endoscopy* , reporting on interesting cases and new techniques in gastroenterological endoscopy. All papers include a high-quality video and are published with a Creative Commons CC-BY license. Endoscopy E-Videos qualify for HINARI discounts and waivers and eligibility is automatically checked during the submission process. We grant 100% waivers to articles whose corresponding authors are based in Group A countries and 50% waivers to those who are based in Group B countries as classified by Research4Life (see: https://www.research4life.org/access/eligibility/ ). This section has its own submission website at https://mc.manuscriptcentral.com/e-videos .
